# Human papillomavirus E7 induces p63 expression to modulate DNA damage response

**DOI:** 10.1038/s41419-017-0149-6

**Published:** 2018-01-26

**Authors:** Sahar Eldakhakhny, Qing Zhou, Emma J. Crosbie, Berna S. Sayan

**Affiliations:** Division of Cancer Sciences, School of Medical Sciences, Faculty of Biology, Medicine and Health, University of Manchester, Manchester Academic Health Science Centre, Manchester Cancer Research Centre, Wilmslow Road, Manchester, M20 4QL UK

## Abstract

Cervical cancer is the third most common malignancy diagnosed in women worldwide. The major aetiological factor underlying the malignant transformation of cervical cells is the persistent infection with high-risk human papillomaviruses (HR-HPV), with more than 99% of cases expressing viral sequences. Here, we report a previously unknown mechanism driven by high-risk human papillomavirus E7 protein to modulate response to DNA damage in cervical cancer cells. Our data shows that HR-HPV E7 oncoprotein induces the transcription of the p53-family member p63, which modulates DNA damage response pathways, to facilitate repair of DNA damage. Based on our findings, we proposed a model, where HR-HPV could interfere with the sensitivity of transformed cells to radiation therapy by modulating DNA damage repair efficiency. Importantly, we have shown for the first time a critical role for p63 in response to DNA damage in cervical cancer cells.

## Introduction

Cervical cancer is the third most common malignancy and the fourth leading cause of cancer-deaths among women, with less than a 50% 5-year survival rate in poor resource settings^1–3^. The major aetiological factor underlying the malignant transformation is the persistent infection with high-risk human papillomaviruses (HR-HPV), with more than 99% of cases expressing viral sequences^2,4^.

HPVs are a heterogeneous family of double-stranded DNA viruses with more than 150 different types identified so far^5^. Although they all show tropism to cutaneous or mucosal epithelial cells, approximately one-third specifically infect the genital tract^6,7^. These genital HPVs are further divided into low-risk (LR) and HR groups according to the susceptibility of the induced lesions to undergo malignant transformation. While LR-HPVs do not cause cancer, HR-HPVs, in particular HPV16 and HPV18, are the most frequently observed types in cervical carcinomas^8^.

HPVs link their life cycle to the proliferation and differentiation dynamics of the host cell. While in normal stratified epithelia the only pool of mitotically active cells is located in the basal and parabasal layers^9^, in HPV-infected epithelial cells at suprabasal layers keep their proliferative capacity^10^. This is mostly achieved by HPV E7 protein, which binds to pRb family members and targets them for degradation, leading to release of E2F transcription factor to drive expression of S phase genes^11^. In the case of persistent infection, when the virus is not cleared by the immune system, HPV genome integrates into host chromosomes. Integration typically results in the increased expression and stability of transcripts encoding the viral oncogenes E6 and E7, which is necessary for the pathogenesis of HPV^12^.

It has been shown in transgenic mouse models that E7 is more potent than E6 in the induction of high-grade cervical dysplasia and invasive cervical malignancies, while E6 can only induce low-grade cervical dysplasia, when expressed alone^13^. This suggests that E7’s main role is to promote carcinogenesis, while E6 predominantly functions to enhance and sustain the E7-induced malignant phenotype—mostly by inducing p53 degradation to inhibit cell death and cell cycle arrest pathways^13–15^. Besides forcing cell cycle progression, E7 contributes to malignant transformation by inducing DNA damage^8,16–18^.

p63 is a member of the p53 family of transcription factors that plays a crucial role in the structure and function of stratified epithelia^19–21^. It promotes proliferation of basal layer stem cells, and at suprabasal layers, p63 levels are down regulated, allowing cells to undergo differentiation^19,22^. In normal cervical epithelium, p63 expression is confined to basal and parabasal layers of ectocervix and basal and subcolumnar cells of the cervical transformation zone^23,24^. In mild dysplasia (cervical intraepithelial neoplasia, CIN1) it is expressed in basal and parabasal layers, extending into the middle and upper layers in moderate and severe dysplasia (CIN2 and CIN3)^23,25^.

Although the impact of p63 in the life cycle of HPV has been investigated extensively by over-expressing E6/E7 or the virus itself as an episome in primary keratinocytes^26–28^, there is a knowledge gap regarding the function of p63 in cervical cancer and whether there is an interplay between E6/E7 proteins and p63 during the maintenance of malignant phenotype. Here we report a novel HR-HPV E7 oncoprotein-driven signalling pathway in cervical cancer cells that is mediated by p63, which facilitates repair of DNA damage induced endogenously by the viral oncogenes and exogenously by gamma irradiation. Interestingly, while p63 is rapidly degraded in response DNA damage in keratinocytes and HNSCC cells^29,30^, it is protected from degradation in cervical cancer cells. Our data suggest that induction of p63 expression by E7 could be the underlying factor that confers resistance to cervical cancer cells against radiotherapy. Targeting E7-p63 signalling network may, therefore, offer novel therapeutic approaches to interrupt the DNA repair capacity in cervical cancer cells to overcome radioresistance.

## Results

### E7, but not E6 promotes p63 expression in cervical cancer cells

**Fig. 1:**
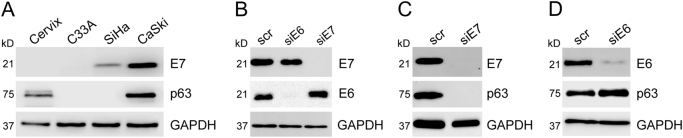
HPV-16 E7 oncoprotein drives p63 expression in cervical cancer cell lines **a** Total protein was extracted from normal cervix tissue, HPV-negative cervical cancer cell line C33A and two HPV16-positive cervical cancer cell lines SiHa and CaSki. Expression levels of E7 and p63 were assessed by western blotting. **b** Western blot analysis of E6 and E7 was carried out 48 h after transfection in CaSki cells to test the specificity and efficiency of the siRNAs. **c and d** p63 protein levels were measured 48 h following E7-depletion **c** or E6-depletion **d** in CaSki cells by western blotting. In all western blots GAPDH was used as equal loading control

Based on their potent roles on the proliferation of cells at stratified epithelia^31–33^, we asked whether expression of E7 has an impact on the expression of p63 or visa versa. To this end, first we assessed the expression profile of p63 in a panel of cervical cancer cell lines and compared this to that of E7. Normal cervical tissue expressed low levels of p63 and as expected did not express E7 protein (Fig. 1a). p63 expression was lost in the p53 mutant HPV-negative cervical carcinoma cell line C33A, was undetectable in SiHa cells, which contain only 1–2 copies of integrated HPV-16 DNA and was very high in CaSki cells, which contain around 600 copies of integrated HPV-16 DNA. These data suggest that malignant transformation of cervical cells may lead to loss of p63 expression, while presence of HR-HPV may recover p63 expression, depending on the integrated copy number of the virus.

This led us to investigate whether expression of E7 has an impact on the expression of p63 in cervical cancer cells. We first analysed the specificity and efficiency of E7 silencing in CaSki cells by transiently transfecting them with siRNA targeting E7 or E6. Our results demonstrated that we could indeed achieve efficient and specific silencing of HPV-16 E6 and E7 expression by RNA interference (Fig. 1b).

Western blot analysis revealed a remarkable reduction in p63 protein levels following transfection of CaSki cells with E7 siRNA, E6-depletion did not affect p63 levels (Fig. 1c and d). These data demonstrate that in CaSki cells, expression of p63 is E7 dependent.

### E7 promotes p63 expression predominantly at transcriptional level

E7 has the ability to alter the transcriptome, proteome, and metabolome of a cell by modulating the expression, localisation, and activity of key regulators within complex signalling networks^34^. To achieve this, it directly interacts with the target protein to modulate its stability^35^ or its activity^36^ or it indirectly modulates its targets by changing cellular phosphorylation profile, causing epigenetic reprogramming or altering the microRNA transcriptome^37–41^.

**Fig. 2:**
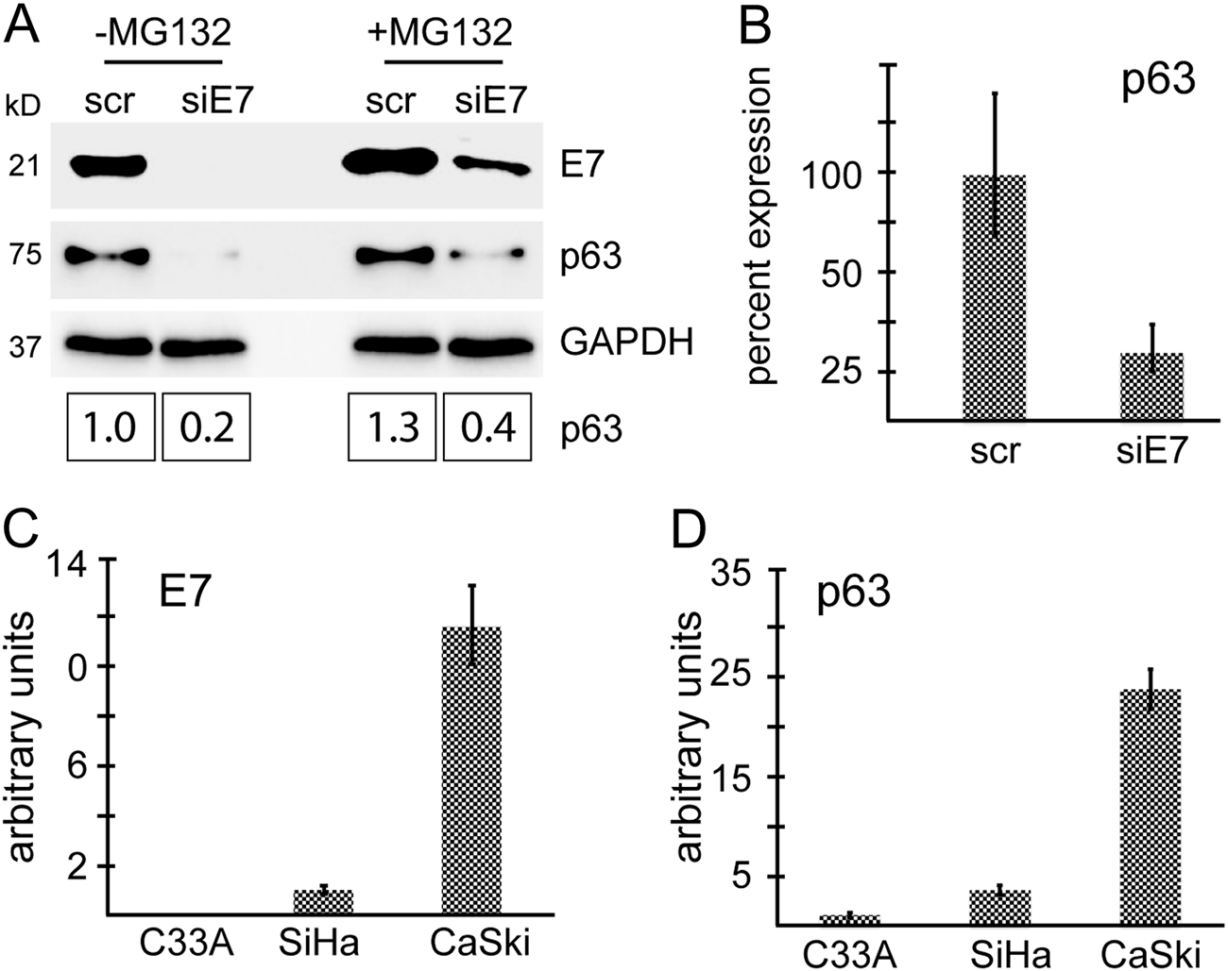
E7 promotes p63 expression at transcriptional level **a** CaSki cells were transfected with scrambled siRNA or E7-siRNA. Forty-eight hours later, cells were treated with the proteasome inhibitor MG132 (20 μM). The untreated control cells and MG132-treated cells were harvested 4 h later and expression of E7 and p63 were assessed by western blotting. The intensity of the bands was quantified by using the ImageJ software. **b** Q-PCR was used to quantitate E7 and p63 transcript levels in E7-depleted cells 48 h following transfection. **c and d** Total RNA from C33A, SiHa, and CaSki cells was extracted and reversed by using random primers. The cDNA was used to analyse the transcript levels of E7 (**c**) and p63 (**d**) by Q-PCR

To gain insight into the mechanisms of induction of p63 expression by E7, we first tested whether E7 enhances the stability of p63, by comparing p63 expression in E7-silenced cells in the presence or absence of the proteasome inhibitor, MG132 (Fig. 2a). Inhibition of proteasome activity resulted in increased E7 and p63 protein levels as described previously^19,42–44^. p63 protein levels were approximately two-fold higher in MG132-treated E7-depleted cells compared to their untreated counterparts, although this was potentially due to persistent expression of E7 despite RNA interference (Fig. 2a).

Then we analysed p63 transcript levels in CaSki cells transfected with siE7. Loss of E7 expression led to a significant reduction in ΔNp63 (referred as to p63 hereafter) mRNA levels (Fig. 2b); which is the only detectable p63 isoform expressed in CaSki cells (Supplementary Fig. 1Sa). This data suggests that E7-mediated p63 expression is regulated predominantly at transcript level. Indeed, comparison of p63 mRNA levels in cervical cancer cell lines also confirmed a correlation between E7 expression and p63 transcript levels (Fig. 2c and d).

It has been shown in differentiating human foreskin keratinocytes that expression of HPV-31 E7 down-regulates miR-203, which leads to accumulation of p63^26^. To investigate the potential contribution of E7-miR-203 axis to p63 expression in CaSki cells, we assessed miR-203 levels in E7-depleted cells by Q-PCR. Interestingly, E7-depletion led to reduced expression of miR-203, suggesting that in HPV-transformed cervical cells, E7-miR-203-p63 axis is not functional (Supplementary Fig. 1Sb).

### p63 overexpression cannot rescue E7-induced cell cycle arrest

**Fig. 3:**
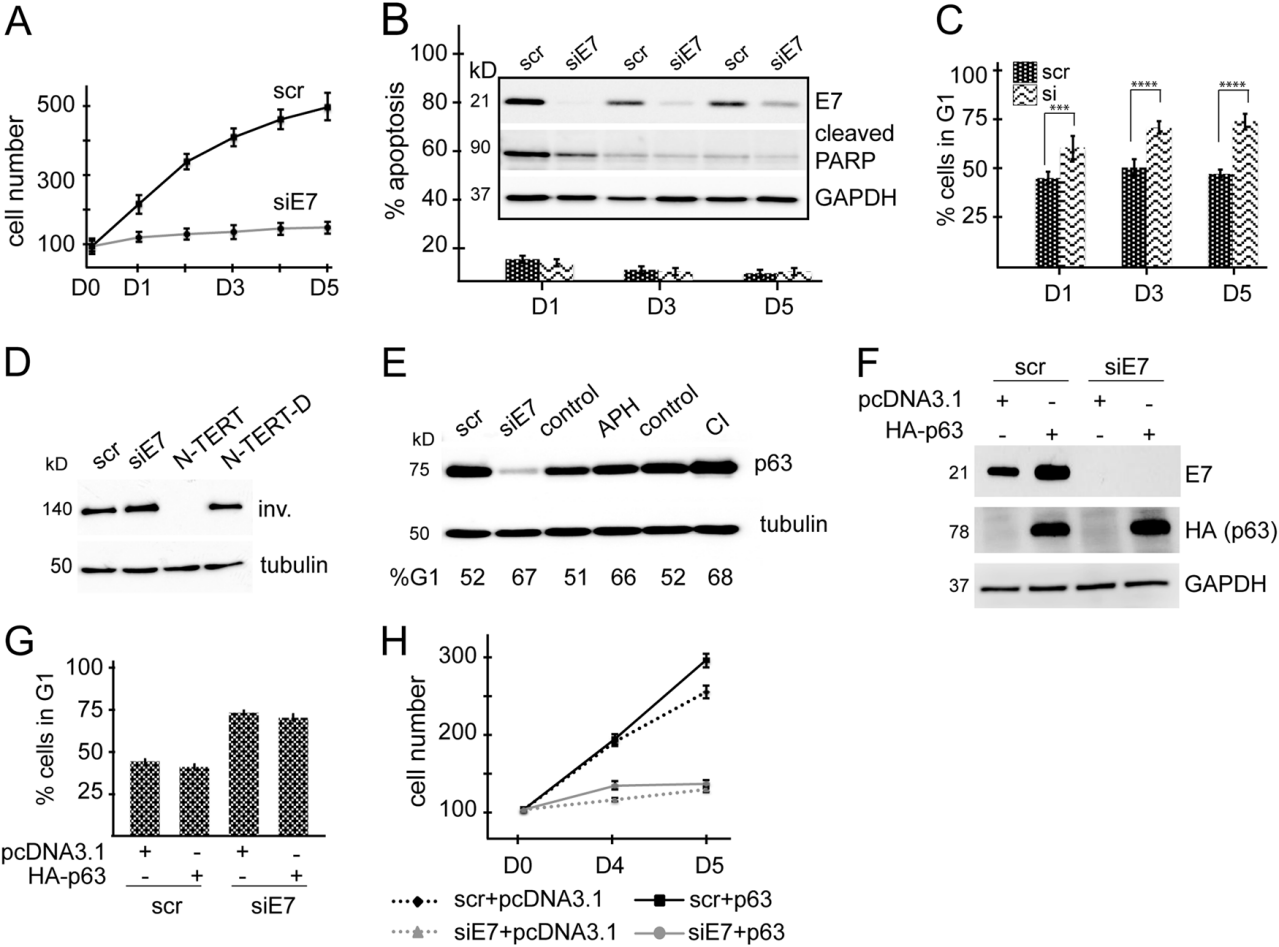
p63 overexpression cannot rescue E7-induced cell cycle arrest **a** CaSki cells were transiently transfected with si-E7 and cell proliferation was assessed at indicated time points. The graph shows the average number of cells in triplicate 24 wells as compared to scrambled-transfected controls. **b** Cell death following E7-depletion was assessed by flow cytometry as described in section “Materials and methods” and also by western blot analysis for cleaved PARP. **c** E7-depletion results in a significant increase in the number of cells undergoing G1-arrest as detected by flow cytometry. Cells were collected at indicated time points following transfection of CaSki cells with siE7 or scrambled control siRNA (scr). Graph shows percentage of cells in G1 following E7-depletion at indicated time points (***P-value < 0.001, ****P-value < 0.0001 calculated by Student’s *t*-test). **d** CaSki cells were transfected as indicated and collected after 5 days. N-TERT cells were induced to differentiate by adding 1.2 mm Ca^2+^ and were harvested after 7 days. Cell lysates from scr/siE7-transfected cells and proliferating and differentiated N-TERT cells were analysed for the expression of the differentiation marker involucrin (inv). **e** G1 arrest was induced in CaSki cells by treating with 6 μM aphidicolin (APH) or by contact inhibition (CI) (growing cells at confluency for up to 7 days). p63 expression was assessed by western blotting. **f** CaSki cells were transfected with siE7 and scrambled control siRNA in duplicates. Twenty-four hours later one plate of cells from each duplicate was re-transfected with pcDNA3.1 empty vector and the other plate of cells with HAp63-pcDNA3.1 plasmid. Cells were collected 48 h later and expression of E7 and p63 was revealed by western blotting. **g** A fraction of cells was used to assess cell cycle profile by flow cytometry. Amount of cells in G1 was quantified and plotted on a graph to demonstrate impact of p63 overexpression on cell cycle. **h** Proliferation assay showing the effect of p63 over-expression in E7-depleted cells compared to scrambled siRNA and/or pcDNA3.1 empty vector transfected control cells. (Error bars represent s.e.m. in all graphs.)

Induction of p63 expression by E7 raised the possibility that p63 may function downstream of E7 to promote cervical cancer cell proliferation. To assess this, we first analysed the consequences of E7 silencing in CaSki cells. Transient silencing of E7 resulted in substantial reduction in cell proliferation (Fig. 3a). This effect was not due to induction of apoptosis in the absence of E7 expression, but rather due to an increased fraction of cells undergoing G1 arrest (Fig. 3b, c and Supplementary Fig. 2Sa). We detected cleaved PARP in both scrambled and siE7-transfected cells 24 h after transfection, which was potentially due to the toxic effect of transfection reagents at this early time point of the experiment.

In stratified epithelia, loss of proliferative potential is often associated with induction of differentiation. To assess if the increase in G1-arrested cell population in E7-depleted cells was due to differentiation, we analysed the levels of the differentiation marker involucrin following transfection of CaSki cells with siE7. As shown in Fig. 3d and Supplementary Fig. 2Sb, we detected high levels of involucrin in CaSki cells and depletion of E7 did not have a significant effect on its expression.

As p63 expression is closely linked to proliferation of keratinocytes, we tested the possibility that loss of p63 in E7-depleted cells could be a consequence of impaired cell proliferation. Here, we induced G1 arrest in CaSki cells by aphidicolin or by contact inhibition. Both approaches induced similar levels of G1 arrest as E7-depletion (Supplementary Fig. 2Sc and d), however they did not induce loss of p63 protein (Fig. 3e).

Finally, to investigate if the impact of E7 on cell proliferation could be rescued by overexpressing p63, we co-transfected CaSki cells with E7 siRNA and HA-p63 (Fig. 3f). Cell cycle analysis revealed that p63 over-expression in E7-depleted CaSki cells was insufficient to restore normal cell cycle progression (Fig. 3g and Supplementary Fig. 2Se). Indeed, comparison of proliferation of p63 over-expressing cells following E7 depletion showed that their proliferation rate was very similar to E7-depleted cells (Fig. 3h).

### p63 is dispensable for cervical cancer cell proliferation

**Fig. 4:**
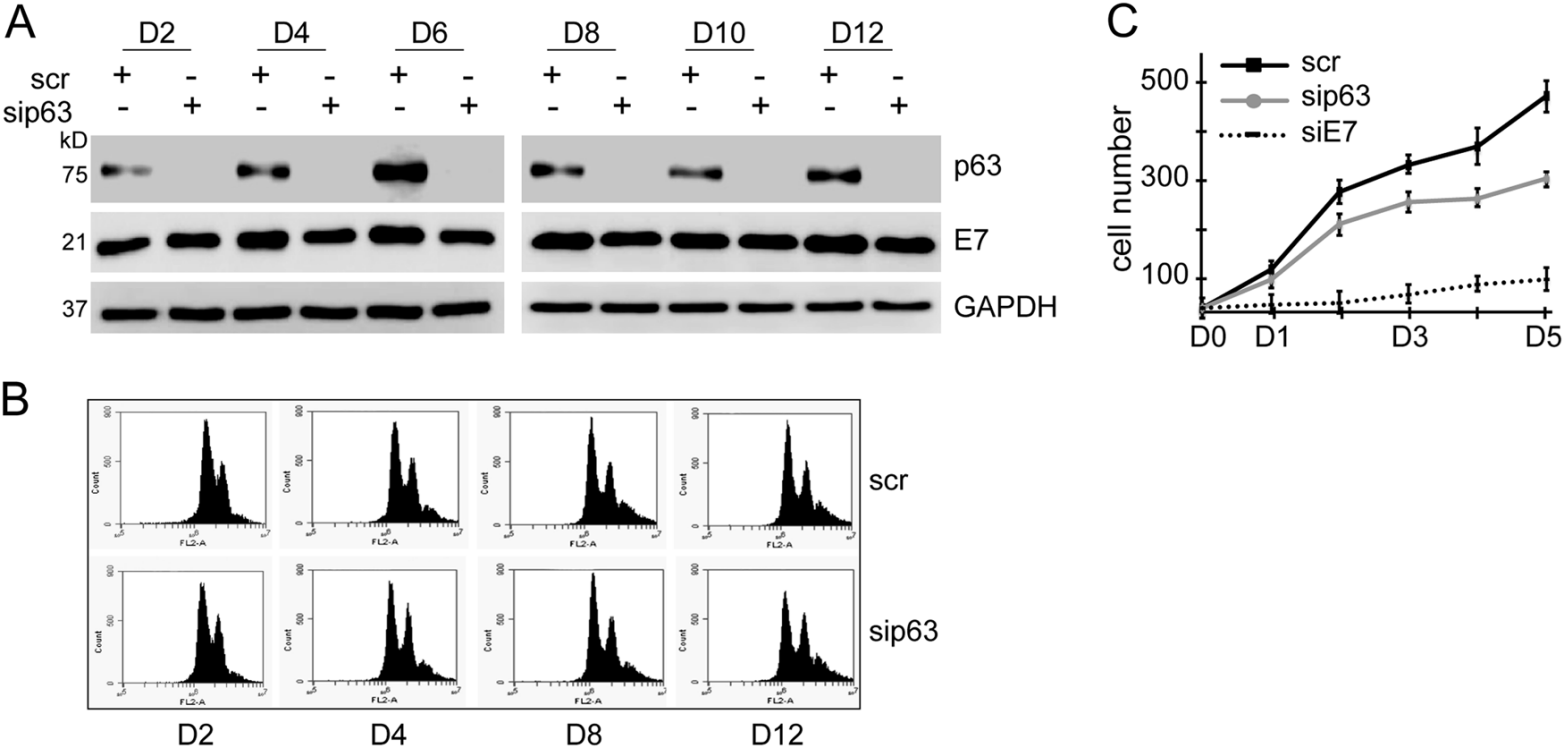
p63 is dispensable for cervical cancer cell proliferation **a** CaSki cells were transfected with p63 siRNA and efficiency of silencing was measured by western blot analysis for up to 12 days. Efficiency of p63 silencing was maintained by re-transfecting the cells 6 days after the initial transfection. Loss of p63 did not have an effect on E7 expression. **b** Proliferation assay was performed to compare the proliferation rate of cells transfected with scrambled siRNA control, siE7, and sip63. The graph shows the average number of cells in triplicate 24 wells. **c** Cell cycle analysis of p63-depleted CaSki cells showing that loss of p63 does not have a significant effect on cell cycle profile in CaSki cells

To further question the possible contribution of p63 to cervical cancer cell proliferation, we silenced p63 expression by RNA interference in CaSki cells and analysed the cell cycle and proliferation profile up to 12 days following transfection. p63 silencing was maintained at high efficiency by re-transfecting the cells 6 days after the initial transfection (Fig. 4a). Unlike E7-depletion, loss of p63 did not cause a significant change in cell cycle profile as assessed by flow cytometry, although a subtle increase in G2/M population was observed 12 days after p63-depletion (Fig. 4b). This finding was also verified by performing a proliferation assay, where sip63-transfected cells proliferated in a very similar manner to scrambled-transfected cells, demonstrating that p63 is not critical for CaSki cell proliferation (Fig. 4c).

### p63 modulates DDR in response to γ-irradiation-induced DNA damage

**Fig. 5:**
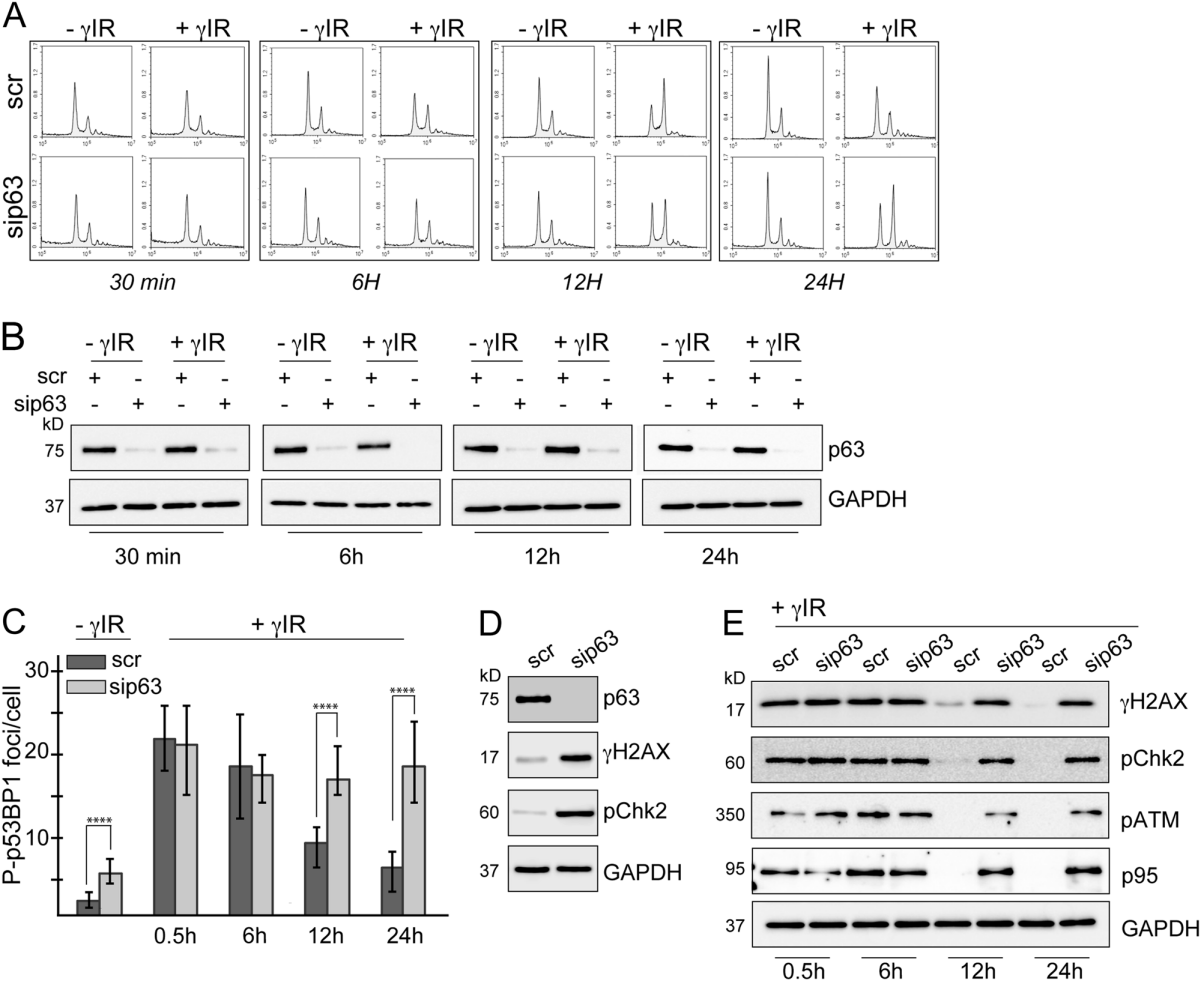
p63 expression modulates DDR in cervical cancer cells CaSki cells were transfected with scrambled or p63 siRNA and exposed to 3.5 Gy ionising radiation 48 h following transfection. Cells were collected at indicated time points post-irradiation. **a** Cell cycle analysis showing that in the absence of p63, CaSki cells fail to undergo G2/M arrest in response the gamma irradiation at earlier time points. **b** Western blot analysis of p63 expression in irradiated cells and the untreated controls. **c** A fraction of cells were fixed and subjected to immunocytochemical staining for phospho-p53BP1. The number of foci per nucleus was quantified by using ImageJ. Error bars represent the s.e.m. ****p < 0.0001. **d** Western blot analysis showing the levels of γH2AX and pChk2 in scrambled and p63 siRNA-transfected CaSki cells, 48 h after transfection. **e** Activation of DDR following was assessed by measuring the levels of γH2AX and pChk2 by western blotting

DDR involves a series of tightly regulated processes that coordinate chromatin organisation and cell cycle progression with genome surveillance and repair mechanisms^45^. When DNA damage is sensed, it initially ensures that the cells temporarily stop cell cycle progression, allowing the repair of the lesions. Therefore, we first analysed how loss of p63 affects cell cycle dynamics in CaSki cells upon induction of DNA damage by γIR (Fig. 5a). p63 expressing cells started to undergo G2/M arrest 6 h after irradiation, had the highest fraction of G2/M arrested cells at 12 h time point and returned back to normal G1/S/G2 distribution 24 h after γIR. On the other hand, p63-depleted cells showed a slightly delayed and significantly prolonged response to γIR, with a subtle G2/M arrest after 6 h and a high fraction of G2/M arrested cells after 24 h (Fig. 5a). Unlike keratinocytes and head and neck squamous carcinoma (HNSCC) cells^46^, where p63 is rapidly degraded following damage, in CaSki cells DNA damage did not induce p63 degradation (Fig. 5b). Expression of TA and ΔNp63 isoforms was also not changed at transcript level upon γIR (Supplementary Fig. 3S).

To have an insight into the dynamics of DDR in the presence or absence of p63, we analysed phosphorylation of p53BP1 and H2AX by immunofluorescence. Absence of p63 led to a small but significant increase in phosphorylation of p53BP1 in the absence of induced-DNA damage (Fig. 5c and Supplementary Fig. 4Sa). At early time points following exposure to γIR, number of phospho-p53BP1 foci increased at comparable levels in p63 expressing cells and their p63-depleted counterparts. However after the 6 h time point, number of foci started to decrease steadily only in the cells that express p63, while p63-depleted cells maintained high levels of foci at later time points. Phosphorylation of H2AX followed a very similar pattern to that of p53BP (Supplementary Fig. 4Sb).

Western blot analysis confirmed that silencing of p63 alone is sufficient to induce H2AX phosphorylation and downstream activation of Chk2, although this does not result in cell cycle arrest (Fig. 5a–d). This is potentially because the un-repaired endogenous damage is not substantial enough to trigger a detectable cell cycle arrest.

Assessment of activation of Chk2, ATM, and p95 in p63-expressing and depleted cells following γIR confirmed that loss of p63 impairs DDR. Similar to γH2AX, pChk2, ATM, and p95 levels increased at similar levels in both cell groups up to 6 h and then started to show significant differences at late time points; p63-depleted cells still exhibiting high levels, 24 h after γIR (Fig. 5e). These results suggest that the role of p63 downstream of E7 in HPV transformed cells is to facilitate DNA damage repair.

As a transcription factor, p63 mediates most of its downstream functions by transcriptionally activating/inactivating target genes. It has been shown that p63 knock out mouse embryonic fibroblasts (MEFs) show enhanced radiosensitivity to γIR as they fail to repair double-strand breaks (DSBs). This is mostly attributed to the ability of p63—in particular—p63 to induce key regulators of DSB repair, including BRCA2, Mre11, Rad50, and Rad51 in MEFs^47^. To have insight into the molecular basis of regulation of DDR by p63, we analysed p63-transcriptional targets for DDR-specific genes and identified that it up-regulates ATM, RPA1, BRCA2, and RAD51AP^31,48^. Analysis of expression levels of these genes in scrambled siRNA-transfected and sip63-transfected CaSki cells revealed that p63 modulates the cellular levels of these DDR regulators (Supplementary Fig. 4Sc).

### HR-HPV E7 is more potent than LR-HPV-E7 in promoting p63 expression

**Fig. 6:**
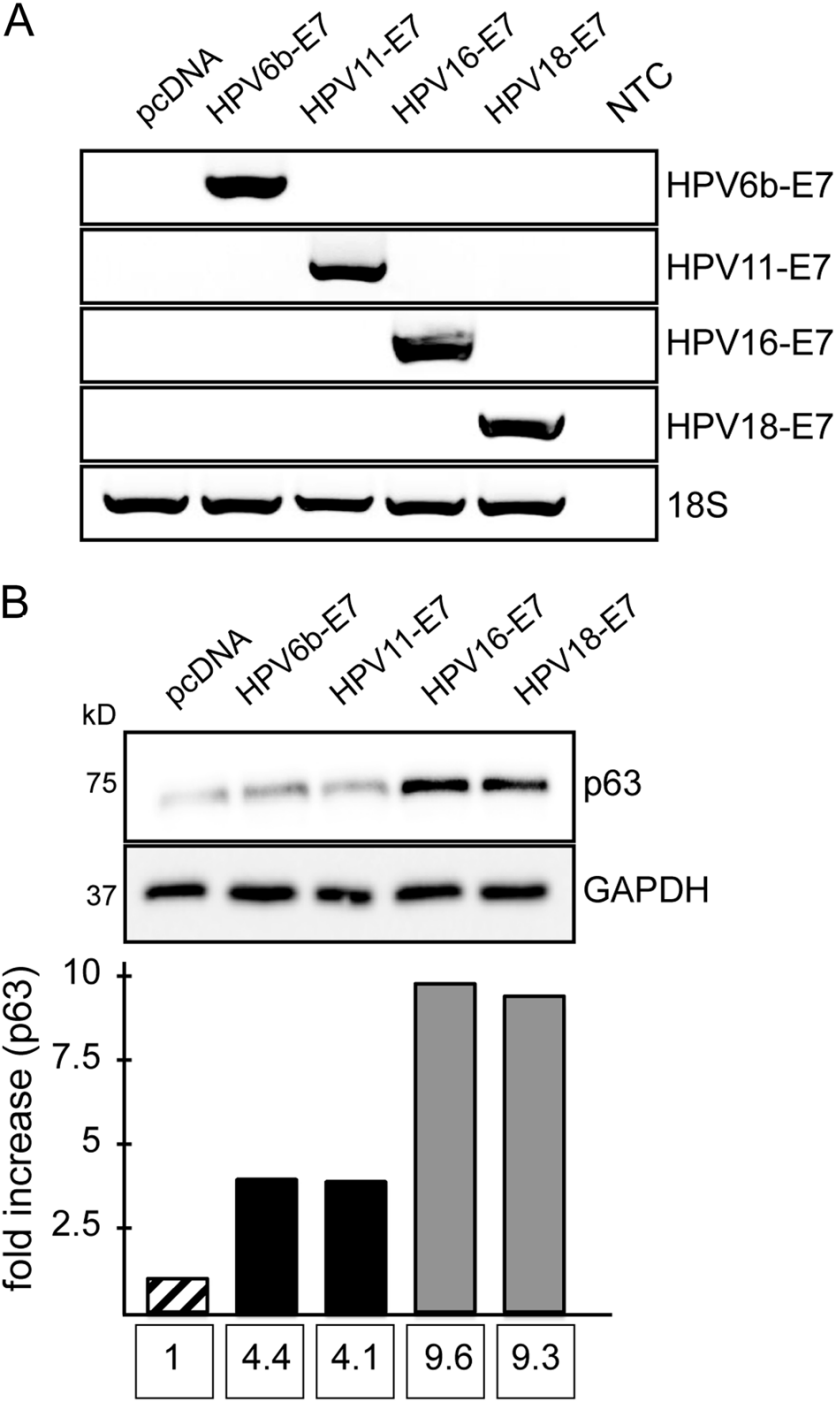
HR-HPV E7 is more potent than LR-HPV E7 in promoting p63 expression N-TERT cells were transfected with plasmids encoding HPV-6b E7, HPV-11 E7, HPV-16 E7, and HPV-18 E7. Cells were collected 5 days after transfection for analysis. **a** All plasmids expressed comparable levels of E7 as detected by semi-quantitative RT-PCR. **b** Western blot analysis of p63 following E7 overexpression. Bands were quantified by using the ImageJ software

The striking effect of p63 on the response of cervical cancer cells to DNA damage led us to investigate whether induction of p63 expression by E7 protein could be different between HR and LR-HPV. To this end, we over-expressed LR-HPV (6b and 11) and HR-HPV (16 and 18) E7 in N-TERT cells and analysed their impact on p63 protein expression. All vectors expressed E7 at comparable levels (Fig. 6a). Analysis of p63 expression revealed that HR-HPV E7 could induce p63 expression at least two-fold more than LR-HPV E7, suggesting that this could be a critical step in the induction of malignant phenotype by the HR virus (Fig. 6b).

## Discussion

HPVs infect cells at the basal layer of stratified epithelia, where the p53 family protein p63 is highly expressed and is essential for proliferation^49,50^. Although in normal stratified epithelia the only pool of mitotically active cells is located in the basal and parabasal layers^9^, following HPV infection, cells at suprabasal layers keep their proliferative capacity to replicate the HPV genome^8,38^. This is mostly achieved by HPV E7 protein, which forces cell cycle progression by inducing degradation of pRb^17^. In moderate and severe cervical dysplasia, where cells at suprabasal layers still undergo proliferation, p63 is expressed throughout the stratified epithelia^23,25^, suggesting a possible relationship between the expression of E7 and p63.

Indeed, comparison of the expression levels of E7 and p63 in a panel of cervical cancer cell lines and normal cervix tissue (Fig. 1a) revealed that in HPV-positive cell lines SiHa and CaSki, expression profile of p63 showed marked similarities to that of E7, and silencing of E7 resulted in a substantial decrease in p63 protein levels (Fig. 1). Unexpectedly, p63 was reduced in C33A cell line, compared to normal cervix. C33A has recently been shown to be a metastatic cell line that forms lymph node metastases following subcutaneous implantation in immunocompromised mice^51^. This may suggest that loss of p63 expression could be a pre-requisite for HPV-negative cervical cancer cell metastasis, as previously described for prostate cancer^52^.

Analysis of E7-depleted CaSki cells revealed a prominent relationship between E7 expression and p63 transcript level (Fig. 2). E7 could potentially regulate p63 transcript levels either by inhibiting the expression of p63-targeting microRNAs or by activating de novo transcription by binding to, and activating transcriptional regulators of p63. miR-203 is expressed at high levels in the suprabasal layers of stratified epithelia, where it targets p63 expression in order to inhibit proliferation and promote differentiation^53^. As HPV-31 E7 has been shown to inhibit miR-203 expression in keratinocytes^26^, we investigated a potential link between E7 and miR-203 in CaSki cells. Interestingly, our results showed that E7 depletion led to a decrease in miR-203 levels, rather than an increase as was shown in keratinocytes (Supplementary Fig. 1Sb). It has been shown in cervical tissues and cervical cancer that miR-203 and p63 co-exist and display a similar expression pattern^54^. This suggests the existence of more complicated regulatory pathways in the cervix and in cervical cancer. Activation of de novo p63 transcription through interaction of E7 with p63-transactivating transcription factors is another possible mechanism that could regulate p63 levels in HPV-transformed cells. For instance, E7 has been shown to interact with AP-1 and forkhead box transcription factors^55^, both of which has binding sites on p63 promoter (data not shown).

As both E7 and p63 are important for cell proliferation in stratified epithelia, we investigated whether p63 functions downstream of E7 to promote proliferation. Depletion of E7 expression resulted in inhibition of cell proliferation due to an increase in the number of cells undergoing G1-arrest and loss of p63 under these circumstances was not due to impaired cell proliferation (Fig. 3). Over-expression of p63 in these cells was not sufficient to rescue cells from G1 arrest. In fact, depletion of p63 expression in CaSki cells did not affect the proliferation rate significantly, demonstrating that p63 is not essential for the proliferation of cervical cancer cells and that other E7-driven signalling pathways are much more effective than p63-driven pathways (Fig. 4).

HPV induces DDR in infected cells both during the early stages of the infection as an episome (due to the unusual structure and replication dynamics of the viral genome) and following insertion of viral oncogenes into the host chromosomes (due to expression of E6 and E7)^56–59^. As p63 is also known to modulate DDR pathway^47,60^, we investigated a potential role for p63, acting downstream of E7, in the regulation of DNA damage response. Our results demonstrated that p63 expression is essential for the repair of DNA lesions that are induced endogenously (by viral oncoprotein expression) and exogenously (by γIR) (Fig. 5). Depletion of p63 expression results in an increase in phosphorylation of H2AX, p53BP1, and Chk2 in non-irradiated cells, although it does not induce G2/M arrest (Fig. 5d). However, silencing of p63 expression for up to 12 days results in a subtle increase in G2/M population (Fig. 4) associated with gradually increasing H2AX phosphorylation (Supplementary Fig. S5), suggesting that accumulation of endogenous damage will eventually lead to a detectable G2/M arrest. These results also suggest that induction of p63 by E7, could be an important step in adaptation of HPV-infected cells to proliferate in the presence of constant endogenous DNA damage, where p63 induces repair of the damage rapidly, preventing accumulation of damage and thus allowing cells to escape from cell cycle arrest. In the case of extensive DNA damage induced by γIR, the role of p63 in DDR becomes more evident, where p63-depleted cells show signs of impaired DDR signalling, with delayed G2/M arrest and high levels of unrepaired DNA lesions at later time points (Fig. 5). To our knowledge, this is the first time p63 has been shown to be a critical modulator of DDR in cervical cancer.

While infection with HR-HPVs are frequently found in cervical cancers, LR-HPVs are hardly detected in malignancies^61^. If HR-HPV-E7-induced p63 expression were critical for the repair of virus-induced endogenous damage and escape from consequent cell cycle arrest, then we would expect to see a difference between the abilities of HR-E7 and LR-E7 to promote p63 expression. Indeed, overexpression of HPV6b and HPV11 E7 in keratinocytes resulted in only a slight induction in p63 protein levels, while HPV16 and HPV18 E7-induced p63 expression significantly (Fig. 6). This might be due to the differential affinity of LR and HR-HPV E7 protein to certain regulators of p63 transcription, as in the case of pRb, where LR-HPV E7 binds pRb with lower affinity compared to HR-HPV^62,63^.

Radiotherapy relies on production of irreparable DNA damage to induce apoptosis and is the mainstay of treatment for advanced cervical cancer. Its success, however, is hampered by radioresistance, which results in disease recurrence. One of the mechanisms of radioresistance is reduction of radiation toxicity by repair of radiation-induced DNA damage^45,64,65^. As such, significant amount of research is now directed to investigate radioresistance signatures aiming to identify targets for pharmacological inhibition of DNA damage repair pathways to reverse radioresistance^66^. Our findings suggest that HPV-E7-p63 signalling axis could play an important role in resistance to radiation-induced cell death in HPV-transformed cells, and targeting this signalling axis might open-up novel opportunities to tackle radioresistance.

## Materials and methods

### Cell lines and clinical samples

All cell lines were grown as recommended by ATCC. Normal cervical tissue was obtained with ethical approval from The University of Manchester, UK.

### Transfections and treatments

Lipofectamine-2000 (Invitrogen, Thermo Fisher Scientific, Paisley, UK) was used for transfections as suggested by the supplier. Predesigned siRNA targeting p63 was purchased from Thermo Fisher Scientific (pool of #4798, #217143, #115454) (Paisley, UK), targeting HPV-16 E6 and HPV-16 E7 were from Santa Cruz (sc-156008 and sc-270423) (Santa Cruz Inc.). Scrambled control siRNA was from Qiagen (#1027310) (QIAGEN Ltd., Manchester, UK). E7 expression plasmids for LR-HPV6b (#37917) and 11 (#37912), HR-HPV16 (#37880) and 18 (#37886) were obtained from Addgene (Cambridge, USA). MG132 was from Bio-Mol, Aphidicolin was from Sigma Aldrich. 1.2 mM of CaCl_2_ was used to induce differentiation in N-TERT cells.

### Western blot analysis

Cells lysates were prepared by sonicating the cell pellets in lysis buffer (25 mM Tris, 6.4% glycerol, and 0.8% SDS), (Soniprep 150 plus ultrasonic disintegrator (MSE Ltd., Lower Sydenham, London, UK). Protein concentration was measured using Pierce BCA colorimetric assay (Thermo Fisher Scientific, Paisley, UK) according to the manufacturer’s instructions. Equal amounts of total proteins (20–40 μg) were resolved by SDS-polyacrylamide gel electrophoresis, transferred to Amersham Protran Premium nitrocellulose membrane (GE Medical Systems Ltd., Buckinghamshire, UK), probed with the indicated primary antibody and then with HRP-conjugated secondary antibody. Signals were detected by enhanced chemiluminescence and visualised on X-Ray films, LI-COR C-DiGit^®^ Blot Scanner (Cambridge, UK) or Bio-Rad ChemiDoc Imaging System (Hertfordshire, UK). Β-Actin, GAPDH, or α-Tubulin was used as internal control for equal protein loading and analysis.

Primary antibodies used were the following: HPV16 E6/18 E6 C1P5 (sc-460), HPV16 E7 (sc-65711), p63α H-129 (sc-8344), p53 (sc-126), GAPDH (sc-32233), and α-Tubulin (sc-8035), purchased from Santa Cruz Biotechnology (Heidelberg, Germany). p63-α (#13109), Phospho-Chk2 (#2197), Phospho-Histone H2AX (#9718), Phospho-53BP1 (#2675), Phospho-ATM (#5885), Phospho-p95/NBS1 (#3001), and cleaved PARP (#5625) were purchased from Cell Signalling (New England Biolabs (UK) Ltd., Hertfordshire, UK). Involucrin antibody was from Sigma Aldrich (I9018). Secondary goat anti-mouse IgG HRP conjugated (P0447), goat anti-rabbit IgG HRP conjugated (P0448) were both from Dako (Hamburg, Germany).

### RNA isolation and PCR

Total RNA was extracted using RNeasy Plus Mini Kit (Qiagen, UK), as described previously^67^. The quantity of isolated RNA was measured using an ND-2000C spectrophotometer (NanoDrop Technologies, UK). cDNA was synthesised by using 4 μg of total RNA with a RevertAid First Strand cDNA Synthesis Kit (Thermo Scientific, UK) for semi-quantitative PCR analysis.

For semi-quantitative PCR, a 35 cycle PCR was performed for the detection of HPV6b E7, HPV11 E7, HPV16 E7, and HPV18 E7 with the following conditions: denaturation at 94 °C for 30 s, annealing at 55 °C for 30 s, and extension at 72 °C for 30 s. p63 and 18s PCRs were performed for 28 cycles with denaturation at 94 °C for 30 s, annealing at 62 °C for 30 s, and extension at 72 °C for 30 s. The resultant amplified DNA fragments were resolved by electrophoresis on 2% agarose gels containing GelRed Nucleic Acid Gel Stain, 10,000X (Biotium Inc., UK). TA and DN-specific PCRs were done as described previously^19^. Quantitative PCRs were performed with TaqMan Universal Master mix with MJ Research DNA Engine Opticon2 or with QuantStudio-3 Real-Time PCR system. All TaqMan assays were purchased from Applied Biosystems except for the E7 primers, which were purchased from PrimerDesign. For miR-203 detection, TaqMan MicroRNA Reverse Transcription kit and TaqMan universal master mix (Applied Biosystems) were used. Specific primers for miR-203 and RNU6B internal control primers were from Applied Biosystems.

### Immunofluorescence

CaSki cells were plated on glass-coverslips and irradiated with a single exposure of 3.5 Gy by using CellRad by Faxitron. Cells were fixed at indicated time points with 4% paraformaldehyde (EMS, CN Technical Services, UK), permeabilised with 0.1% Triton X-100 (Thermo Fisher Scientific, Paisley, UK), blocked with 3% BSA in PBS-T and incubated with primary antibody at room temperature for 30 min, followed by incubation with Alexa Flour 488-labelled secondary antibody (Thermo Fisher Scientific, Paisley, UK). Finally, the cells were counterstained with DAPI at room temperature for 10 min. ProLong^®^ Gold Antifade reagent (Thermo Fisher Scientific, Paisley, UK) was added and images were acquired by using Olympus IX73 Microscope.

### Cell cycle, proliferation, and apoptosis assays

Cells were counted at indicated time points by using BD Accuri™ C6 flow cytometry (BD Biosciences, UK). For cell cycle and apoptosis assays, cells were harvested, washed in PBS and re-suspended in hypotonic fluorochrome solution, made by diluting 1 mg/ml stock PI solution 1:20 (Sigma, UK) in hypotonic buffer (0.1% (w/v) sodium citrate, 0.1% (v/v) Triton X-100). The suspension was briefly vortexed, stored for 1 h in the dark at 4 °C and analysed by using BD Accuri™ C6 flow cytometry (BD Biosciences, UK) or Novocyte flow cytometer.

## Electronic supplementary material

Supplementary Figure Legends

CaSki cells express ΔNp63, not TAp63 and E7-p63 regulatory axis does not involve microRNA-203

Loss of E7 induces cell cycle arrest

Gamma irradiation does not induce the expression of TAp63 isoform

p63 modulates DDR

Loss of p63 induces DDR
