# Supplementary material for: Human papillomavirus E7 induces p63 expression to modulate DNA damage response
Source: Cell Death Dis. 2018 Jan 26;9(2):127. doi: 10.1038/s41419-017-0149-6 (PMC5833683; doi:10.1038/s41419-017-0149-6)
Supplement: Supplementary file 1 — Supplementary Figure Legends [file 41419_2017_149_MOESM1_ESM.pdf]

SUPPLEMENTARY FIGURE LEGENDS

Supplementary Figure S1: CaSki cells express ΔNp63, not TAp63 and E7-p63 regulatory axis does not involve microRNA-203 (a) Total RNA from CaSki cells, transfected with scrambled siRNA or E7-siRNA and harvested 48H after transfection. Total RNA was extracted and reversed by using random primers. SQ-PCR was used to assess transcript levels of TAp63 and ΔNp63. 18S was used as housekeeping gene to normalize sample-to-sample variations. PCR using TAp63 plasmid or ΔNp63 plasmid as template was used as positive control for each isoform-specific PCR. (scr: scrambled control siRNA, NTC: no template control) **(b)** miR-203 expression in CaSKi cells following E7-depletion was analysed by qRT-PCR.

Supplementary Figure S2: Loss of E7 induces cell cycle arrest (a) CaSki cells were transiently transfected with siE7 and cell cycle was assessed at indicated time points by flow cytometry. **(b)** CaSki cells were transfected as indicated and collected after 5 days. Cell lysates from scr/siE7-transfected cells were analysed for the expression of the differentiation marker involucrin (inv) by qRT-PCR. 18S was used as housekeeping gene to normalize sample-to-sample variations. **(c)** G1/S arrest was induced in CaSki cells by aphidicolin (APH) treatment or by contact inhibition (CI). Cell cycle was analysed by flow cytometry. **(d)** A fraction of cells from (c) was used to quantitate G1/S/G2 populations by flow cytometry and results were plotted on a graph. **(e)** CaSki cells were transfected with siE7 and scrambled control siRNA in duplicates. 24 hours later one plate of cells from each duplicate was re-transfected with pcDNA3.1 empty vector and the other plate of cells with HAp63-pcDNA3.1 plasmid. Cells were collected 48 hours later and analysed for cell cycle profile.

Supplementary Figure S3: Gamma irradiation does not induce the expression of TAp63 isoform. CaSki cells were transfected with scrambled control siRNA or p63 siRNA, and exposed to 3.5Gy ionising radiation 48 hours after transfection. Total RNA was extracted from harvested cells 48H after γIR. SQ-PCR was used to assess transcript levels of TAp63 and ΔNp63. 18S was used as housekeeping gene to normalize sample-to-sample variations. PCR using TAp63 plasmid or ΔNp63 plasmid was used as positive controls for each isoform-specific PCR. (scr: scrambled control siRNA)

Supplementary Figure S4: p63 modulates DDR. p63-expressing and p63-depleted CaSki cells on cover-slips were fixed at indicated times following γIR. Phosphorylation of p53BP1 **(a)** and H2AX **(b)** was analysed by immunofluorescence. Blue: DAPI, Green: pp53BP1 (a)/ γH2AX (b). (Bar: 5 um) **(c)** Q-PCR analysis showing changes in the expression RAD51AP, RPA1, ATM and BRCA2, 48H after p63 depletion in CaSki cells.

Supplementary Figure S5: Loss of p63 induces DDR. CaSki cells were transfected with p63 siRNA and re-transfected 6 days after the initial transfection, to maintain transfection efficiency. Lysates from indicated time points were run on acrylamide gels to analyse γH2AX levels, in order to assess the impact of long-term p63-depletion on the response of cells to intrinsic DNA damage.
