# Supplementary figures and images for: Human papillomavirus E7 induces p63 expression to modulate DNA damage response
Source: Cell Death Dis. 2018 Jan 26;9(2):127. doi: 10.1038/s41419-017-0149-6 (PMC5833683; doi:10.1038/s41419-017-0149-6)

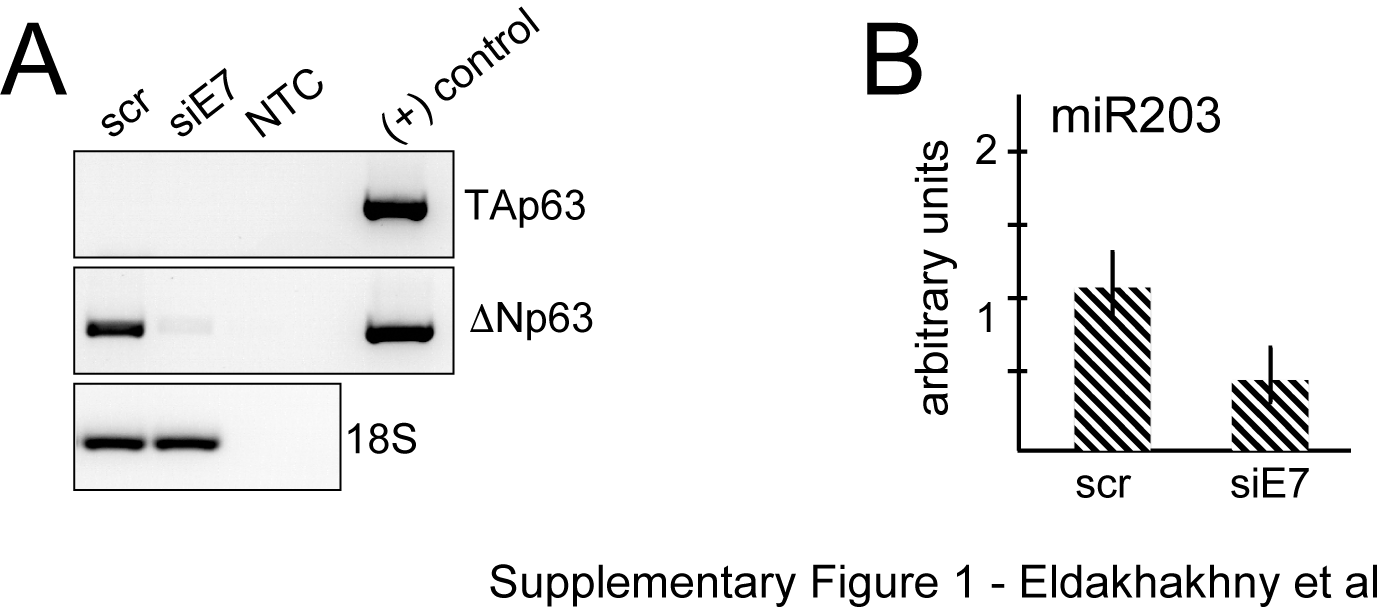

Supplement: Supplementary file 2 — CaSki cells express ΔNp63, not TAp63 and E7-p63 regulatory axis does not involve microRNA-203 [file 41419_2017_149_MOESM2_ESM.tif]

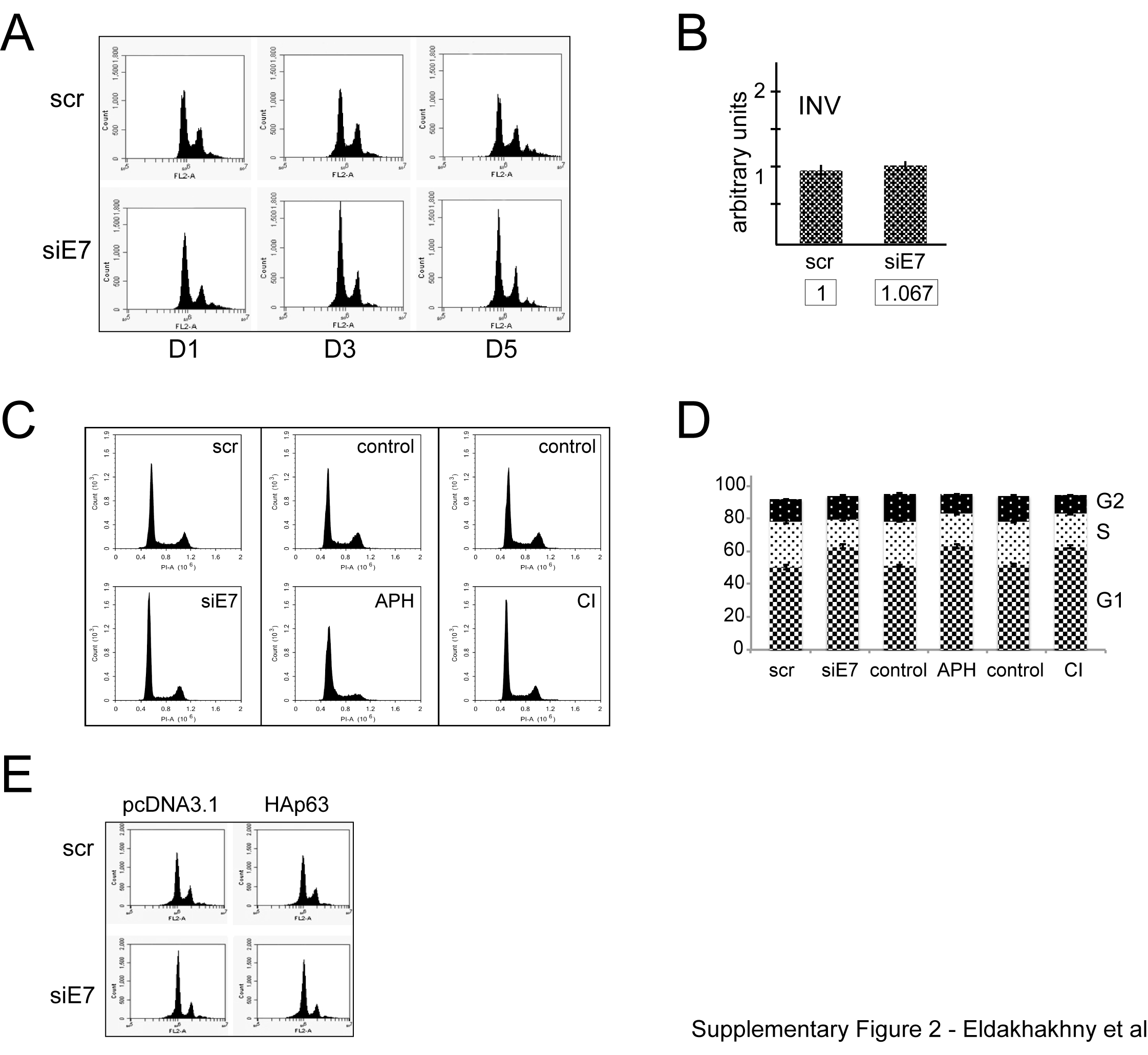

Supplement: Supplementary file 3 — Loss of E7 induces cell cycle arrest [file 41419_2017_149_MOESM3_ESM.tif]

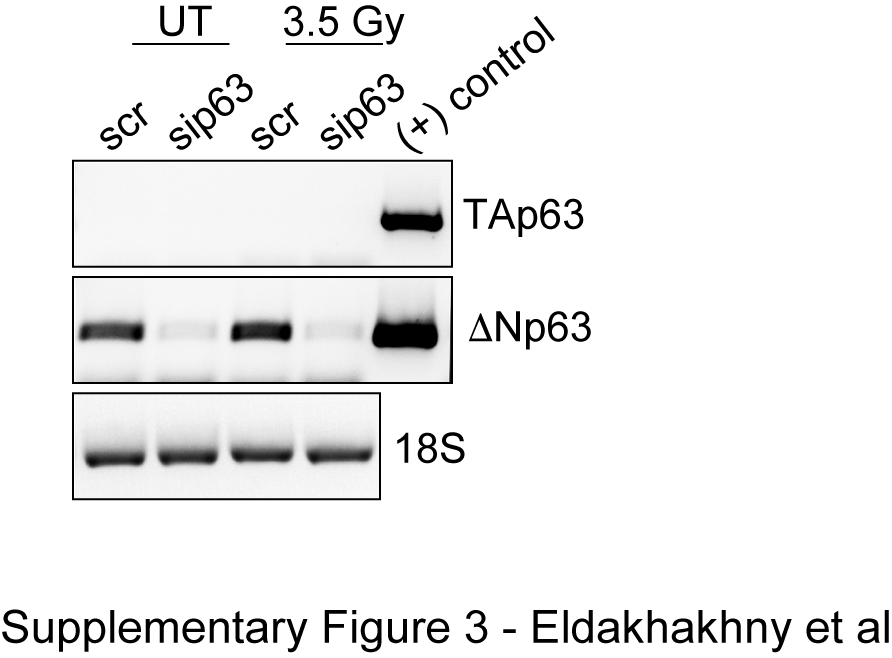

Supplement: Supplementary file 4 — Gamma irradiation does not induce the expression of TAp63 isoform [file 41419_2017_149_MOESM4_ESM.tif]

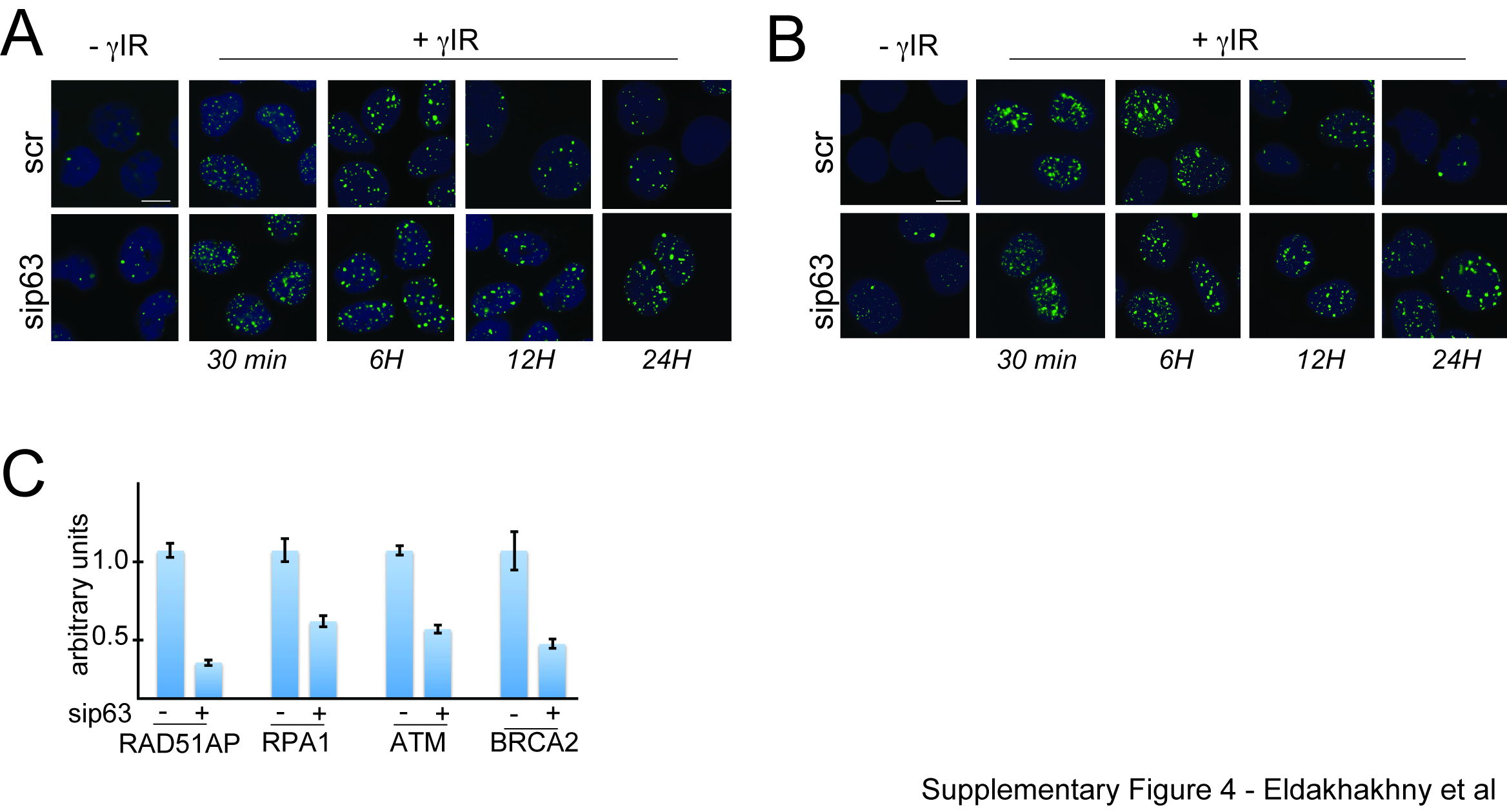

Supplement: Supplementary file 5 — p63 modulates DDR [file 41419_2017_149_MOESM5_ESM.tif]

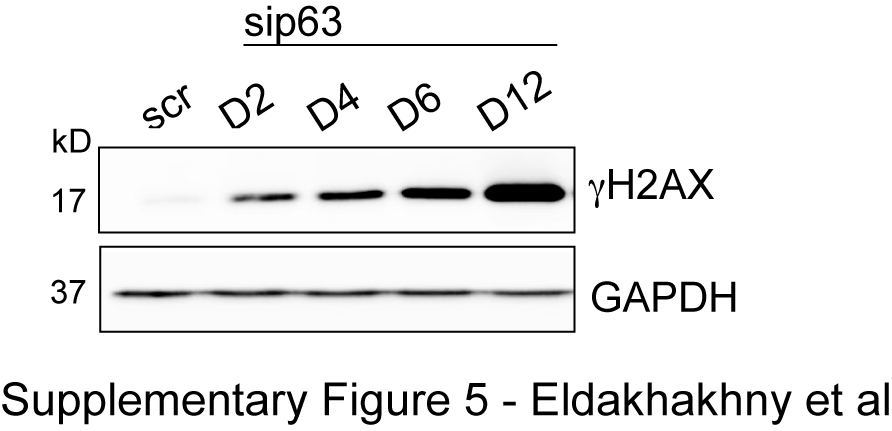

Supplement: Supplementary file 6 — Loss of p63 induces DDR [file 41419_2017_149_MOESM6_ESM.tif]
